# Tet2 at the interface between cancer and immunity

**DOI:** 10.1038/s42003-020-01391-5

**Published:** 2020-11-12

**Authors:** Shuai Jiang

**Affiliations:** Department of Microbiology and Immunology, Louisiana State University Health Sciences Center, Shreveport, LA 71130 USA

**Keywords:** Tumour immunology, Tumour immunology

## Abstract

Keeping a balance between DNA methylation and demethylation balance is central for mammalian development and cell function, particularly in the hematopoietic system. In various mammalian cells, Tet methylcytosine dioxygenase 2 (Tet2) catalyzes oxygen transfer to a methyl group of 5-methylcytosine (5mC), yielding 5-hydroxymethylcytocine (5hmC). Tet2 mutations drive tumorigenesis in several blood cancers as well as in solid cancers. Here I discuss recent studies that elucidate mechanisms and biological consequences of Tet2 dysregulation in blood cancers. I focus on recent findings concerning Tet2 involvement in lymphoid and myeloid cell development and its functional roles, which may be associated with tumorigenesis. I also discuss how Tet2 activities are modulated by microRNAs, metabolites, and other interactors, including vitamin C and 2-hydroxyglutarate (2-HG), and review the clinical relevance and potential therapeutic applications of Tet2 targeting. Finally, I propose key unanswered hypotheses regarding Tet2 in the cancer-immunity cycle.

## Abstract (web summary)

Shuai Jiang discusses the regulation and functional role of the Tet2 enzyme, a regulator of lymphoid and myeloid cell development frequently mutated in haematopoietic cancers. He highlights how Tet2 activity may be modulated to influence cancer immune responses.

## Introduction

DNA methylation/demethylation is dynamically coordinated throughout hematopoietic differentiation, and Tet proteins play crucial roles in adjusting gene expression levels through balancing DNA methylation in hematopoiesis and during immune-cell activation and expansion^1^. The first identified gene of the Tet family, Tet1, acts as a fusion regulator in cases of acute myeloid leukemia (AML) and acute lymphocytic leukemia (ALL)^2^. Two other Tet genes, Tet2 and Tet3, were later identified based on sequence homology with Tet1^3^. In mammalian cells, all Tet family members, Tet1, Tet2, and Tet3, catalyze the successive oxidation of 5mC, yielding 5hmC, 5-formylcytosine (5fC), and 5-carboxylcytosine (5caC)^4^.

**Fig. 1:**
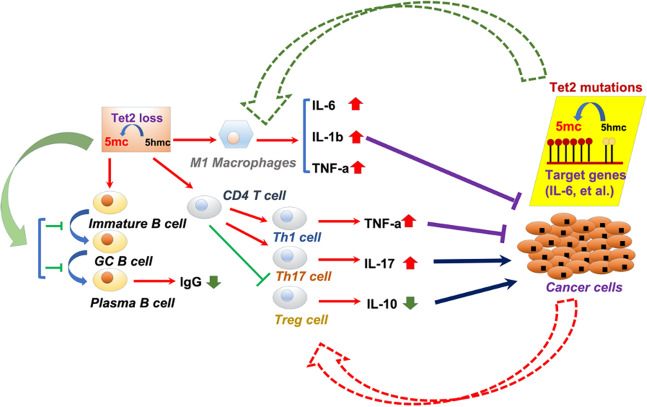
Tet2 in the cancer-immunity crosstalk. Tet2 loss causes an accumulation of 5mC, which promotes B-cell development and B-cell function. Tet2 loss also promotes CD4^+^ T-cell differentiation and M1 macrophage responses, which can modulate cancer cell activities. In contrast, cancer cells that carry Tet2 mutations counteract positive immune-cell responses, including T-cell and macrophages.

The Tet2 gene is subjected to frequent somatic mutations in an extensive range of hematopoietic cancers, including myeloid and lymphoid cancer, and several solid cancers^5,6^. DNA modification by Tet2 is fundamental for gene control in both cancer cells and immune-cell subtypes^5,7,8^. Tet2 protein and its downstream effectors-5hmC/5mC-mediated DNA modification are constitutional in immune cells, including T cells, B cells, and macrophages in both physiological and pathological conditions^9^ (Fig. 1). In blood cancer cells, Tet2 loss is the primary cause of 5mC generation^10^, whereas loss of functional roles of all three Tets is robustly linked to solid cancer progression^11^. Tet2 is one of the commonly mutated genes in hematopoietic cancers^12^. In engineered mouse models, Tet2 knockout (KO), or double KO (DKO) of Tet2 and Tet3, causes myeloid or lymphoid cell spread and the progression of fully infiltrating aggressive tumors^13^.

This review discusses Tet2-related profiles in hematopoietic cancers, different subtypes of adaptive and innate immune cells, and Tet2 regulation by various interactors. I particularly emphasize the potential applications of targeting Tet2 to influence normal and malignant cancer-immunity crosstalk.

## Roles of Tet2 in hematopoiesis, immune-cell lineages, and hematopoietic malignancies

### Tet2 in hematopoiesis

Tet2 epigenetically controls gene expression by modulating methylation-driven gene silencing and is expressed in diverse populations of mouse and human hematopoietic cells^14^. Tet2 mRNA is expressed in hematopoietic cell subpopulations, including progenitors and mature immune cells, displaying detectable levels of 5hmC and 5mC^15^. In mice, Tet2 deletion enhances the Lin^−^Sca-1^+^c-Kit^+^ (LSK) cell subpopulation, which exhibits boosted hematopoietic repopulation competence and biased cell differentiation toward monocyte/macrophage lineages, followed by myeloid cancer progression^16^. In vitro tissue culture work supports that RNA interference (RNAi)-mediated Tet2 silencing in murine hematopoietic precursors can alter their differentiation toward monocyte/macrophage lineages^17^. In mice, Tet2 is pivotal for modulating normal hematopoiesis, and negatively influences hematopoietic stem cell (HSC) homeostasis and differentiation. Tet2 loss reshapes the HSC niche and HSC proliferation/differentiation potential in vitro^14^, whereas Tet2-KO HSCs show elevated ability to reconstitute hematopoiesis in vivo^17^. Tet2 deletion results in reduced genomic 5hmC levels and expands the hematopoietic progenitor cell population in a cell-intrinsic manner^16^. Tet2-KO HSCs are capable of multilineage reconstitution and show a competitive advantage over wild-type HSCs, promoting increased hematopoiesis into myeloid and lymphoid lineages^15^. Tet2 disorder also results in DNA hypermethylation of enhancers in granulocyte–monocyte progenitor (GMP) and embryonic stem (ES) cells^18^. Overall, Tet2 plays vital roles in modulating HSC expansion and function, presumably by controlling 5hmC levels in genes crucial for HSC self-renewal, proliferation, and differentiation, which warrants future examination of the detailed mechanisms.

### Tet2 in myeloid lineages and myeloid malignancies

In humans, Tet2 mutations occur in ~10–30% of myeloid cancers, including myelodysplastic syndromes (MDS), chronic myelomonocytic leukemia (CMML), and myeloproliferative neoplasm (MPN)^19^. Tet2 mutations may be caused by splice site mutations, out-of-frame insertions or deletions and base substitutions. Tet2-KO mice recapitulate characteristics of patients with myeloid cancer, indicating that Tet2 plays a functional role as a tumor suppressor to sustain hematopoietic cell homeostasis. Tet2-KO mice show a phenotype that is similar to CMML, including splenomegaly and enhanced white blood cell (WBC) counts with unequal numbers of monocytes^14^. Moreover, Tet2-KO mice develop an extensive spectrum of myeloid cancers, which occur within the first year of life in ~30% of these animals^17^. Mechanistically, Tet2 blocks leukemic transformation by keeping enhancers free of aberrant DNA methylation that would lead to enhanced stem cell expansion and leukemogenesis^20^. RNAi-mediated Tet2 silencing reduces 5hmC levels in cord blood CD34^+^ cells and promotes progenitor differentiation toward the granulomonocytic lineage^18^. CD34^+^ stem cells from MPN patients with Tet2 mutations reconstitute hematopoiesis in nonobese diabetic/severe combined immunodeficiency (NOD/SCID) mice and display skewed differentiation toward myeloid lineages^15^.

### Tet2 in lymphoid lineages and lymphoid malignancies

Tet2 mutations are also common in both T-cell and B-cell lymphomas, with a 40–70% incidence observed among patients with peripheral T-cell lymphoma (PTCL), particularly in subsets of angioimmunoblastic T-cell lymphoma (AITL)^21^. Adult T-cell leukemia/lymphoma (ATLL) progression accompanies genomic 5hmC loss, suggesting that Tet2 downregulation is the vital mechanism underlying 5hmC regulation during ATLL progression^22^. Tet2-KO mice develop B-cell tumors, and B-cell conditional Tet2 knockout (cKO) mice show B1-cell subset abnormalities and B-cell tumor development after long latency^23^. Tet2 deletion may prompt alterations of peripheral B-cell subsets^23^. Certain Tet2-KO cancers have AID-mediated somatic mutations^23^. Future work is required to investigate whether and how dysregulation of Tet2 impacts on B-cell activation upon various stimuli. Young Tet2-KO mice exhibit a moderately enhanced B1a subset population in the spleen^23^. On the other hand, aging Tet2-KO mice expand clonal CD19^+^B220^low^ immunoglobulin (Ig)M^+^ B-cell subpopulations, which are transplantable and display similarities to chronic lymphocytic leukemia (CLL), including sensitivity to ibrutinib-induced B-cell receptor signaling blockage^24^. Further investigations are needed to examine the molecular signaling pathways controlling B1a-cell development and proliferation in young adult Tet2-KO mice.

In mice, attenuated Tet2 function results in T-cell lymphoma with follicular helper T-cell-like (Tfh) features^25^. Compared with control mice, middle-aged Tet2 gene trap mice (Tet2^gt/gt^) show Tfh-like cell over-expansion in the spleen^25^. Aging Tet2^gt/gt^ mice eventually generate T-cell lymphoma with Tfh-like features^25^. In future research, it will be intriguing to examine how Tet2 works in Tfh cells. A mouse model with conditional Tet2-KO (cKO) in Tfh cells would be valuable for in vivo and in vitro analyses.

Both Tet1 and Tet3 are expressed in hematopoietic cells, without alterations in Tet2-KO cells, demonstrating that Tet1/Tet3 is inadequate to compensate for Tet2 deletion. It will be interesting to dissect their physiological and pathological roles in various immune-cell subsets. Tet2 and Tet3 may cooperate to inhibit abnormal hematopoiesis, including hematopoietic transformation. Further investigations are necessary to test whether and how Tet2 works together with other Tet proteins in distinct blood cancer cells. Deletion of Tet2 in hematopoietic cells or various immune-cell subsets might prompt a specific and progressive enhancer hypermethylation feature linked to altered gene expression, enhanced cell accumulation rate, and leukemia progression, which also warrants future investigation. A better understanding of Tet proteins’ physiological and pathological functional roles may highlight potential novel avenues to develop epigenetic therapies to treat hematological cancers.

Intriguingly, in mice, the combined deletion of Tet1 and Tet2 apparently boosts B-cell cancer, without influencing myeloid cancers^26^. Tet1 and Tet2 are simultaneously downregulated in B-cell acute lymphoblastic leukemia^26^. Although double (DcKO) or triple (TcKO) conditional KO mice would be valuable, it might be challenging to conditionally KO all three Tet proteins in certain immune-cell sub-settings. Identifying Tet2-targeted genes/molecules in myelopoiesis and lymphomagenesis will be crucial for future studies aimed to uncover the molecular mechanisms through which Tet2 modulates hematopoiesis and serves as a tumor-suppressor gene in myelopoiesis and T/B-cell lymphomas. It is intriguing to hypothesize that inactivating somatic Tet2 mutations might latently remain in hematopoietic cells for a long period, possibly even years. Specifically targeting Tet2 or its downstream molecular signaling pathways might offer novel therapeutic strategies for blood cancer prevention.

## Roles of Tet2 in anticancer immune responses

### Tet2 in B-cell and anticancer responses

Tet2 is also essential for B-cell development and function^27^. In mice, Tet2 and Tet3 KO at an early B-cell stage halts the transition of pro-B cells to pre-B cells in the bone marrow (BM), and diminishes the rearrangement of Igk locus^28^. Tet2/Tet3 DKO pro-B cells exhibit enhanced CpG methylation levels at the Ig kappa 3ʹ and distal enhancers^29^. Tet2/Tet3 modulates the kappa-chain expression level, and the DNA modification status of the Ig kappa locus, both in vivo and in vitro^29^. In mice, Tet2 deletion causes myeloid leukemia after a long duration. Compared to Tet2 deficiency, Tet1/Tet2 deletion results in B-cell lymphoma with delayed disease progression^26^. The Tet2/Tet3 deletion in developing B cells leads to B-cell lymphoma development. These mice caused the pathological disorders within six months of age, which is earlier than in Tet1/Tet2-KO mice (~20 months)^7^. Moreover, regulatory B cells (Bregs) play central roles in boosting inflammation and carcinogenesis^30^, but the evidence is lacking regarding whether and how Tet2 protein functions in Bregs both in vitro and in vivo. Continued efforts to investigate the probable anticancer impact of Tet2 in Bregs will guide novel Tet2-based immune therapy. Additionally, in future studies, it would be useful to use B-cell-specific conditional (such as CD19-cKO) Tet2 cKO mice to examine whether and how Tet2 epigenetically affects antibody production in vivo.

### Tet2 in T-cell and anticancer responses

Tet2 deletion augments CD8^+^ T-cell memory differentiation. Deletion of Tet2 advocates early gain of a memory CD8^+^ T-cell fate, in a cell-intrinsic manner, without affecting antigen-driven cell proliferation or effector behavour^31^. After secondary stimulation, Tet2-KO memory CD8^+^ T cells possess remarkable pathogen control capacity, both before and after re-challenge. CD8^+^ T cells with Tet2 conditional KO exhibit full effector functional role upon acute viral infection, and Tet2 deletion enhances memory CD8^+^ T-cell formation^31^. CD8^+^ cytotoxic T lymphocytes (CTLs) are favored immune cells for treating cancer progression^32^. Programmed death‐1 receptor (PD‐1) ligand (PD‐L1) and CTL‐associated antigen 4 (CTLA‐4) are checkpoint receptors that can be targeted to re-activate anticancer CD8^+^ T-cell responses in cancer-immune therapy^33^. It would be interesting to study whether Tet2 might be involved in CTLA-4 or PD-1 pathway-mediated immune checkpoint inhibition for cancer treatment. Further studies are required to explore whether and how Tet2 acts in CD8^+^ CTLs, and how it cooperates with PD-L1/CTLA-4 to modulate anticancer CD8^+^ T-cell responses.

Tet2^flox/flox^CD4^Cre+^ mice have intact thymic and peripheral T-cell subpopulations^31^. Tet2 enhances DNA demethylation and cytokine gene activation, for instance, IL17a, in CD4^+^ T cells. In autoimmune diseases, Tet2 modulates T-cell cytokine production in vivo, and specifically influences IL-10, IFN-g, and IL-17 expression levels^34^. Tet2 depletion in CD4^+^ T cells is linked to reduced cytokine expression, and diminished p300 recruitment^34^. Moreover, Tet2/Tet3 DcKO CD4^+^ T cells induce pathological diseases in healthy mouse^35^. Tet2 enhances typical cytokine gene expression level in Th1 and Th17 cells in vitro, and modulates Th1 and Th17 cell differentiation^34^.

Tet2/Tet3 loss in regulatory T cells (Tregs) contributes to effector behave phenotypes. Treg-cell-specific Tet2/Tet3 double conditional KO mice (Tet2^flox/flox^/Tet3^flox/flox^Foxp3^Cre+^ DcKO mice) show severe inflammation, resulting in inflammatory diseases, and Tregs from these DcKO mice exhibit adjusted expression levels of Treg-typical cytokine genes and dysregulation of genes involved in cancer progression^35^. Overall, these studies reveal that both Tet2 and Tet3 maintain Treg stability and homeostasis. Tet2/Tet3 DcKO in Tregs enhances T-cell activation. Moreover, Tet2/Tet3 DcKO Tregs show an altered cell surface feature and fair hypermethylation of conserved noncoding sequence 2 (CNS2)^35^. Wild-type (WT) Tregs are not able to regulate autoimmunity progression in Tet2-deficient mice^35^. Double Tet1/Tet2 deletion also causes Treg inactivation, differentiation, and even autoimmune diseases^36^. Notably, Tregs hamper anticancer immune surveillance in a healthy person, and prevent effective antitumor immunity in cancer patients, boosting tumor progression^37^. Thus, methods for specific targeting Tet2/Tet3 in Treg cells might be valuable for modulating anticancer immune responses.

### Tet2 in iNKT cell and anticancer responses

Tet2 and Tet3 also work together to restrict invariant natural killer T cells (iNKT cells) proliferation and cell-lineage differentiation^38^. Tet2/Tet3 DcKO mice exhibit impressive iNKT cell proliferation, even at earlier stages. These iNKT cells produce large amounts of cytokines, thus eliciting strong immune responses that prompt other immune-cell subsets responses, and can activate anticancer cytotoxicity^38^. In Tet2/Tet3 DcKO mice, the enhanced iNKT cell numbers and concomitantly higher IL-4 secretion give rise to innate-like CD8^+^ T cells^38^. Tet2/Tet3 deletion yields converted numbers of iNKT cells and significantly influenced gene expression levels, thus changing the identity and functional role of each cell subpopulation^9^. Tet2/Tet3 DcKO iNKT cells show enhanced proliferative abilities, which can explain their in vivo spread^38^. Future immunotherapy methods specifically targeting Tet2/Tet3 to modulate iNKT cell activity could have interdependent consequences and employ strong anticancer immune responses.

### Tet2 in innate immune-cell and anticancer responses

Tet2 is further essential for innate immune-cell functional roles^9^. Compared to other Tets, Tet2 is the most profoundly expressed in murine macrophage differentiation. Tet2 is essential for controlling inflammation through recruiting Hdac2, inhibiting IL-6 levels^40^. Tet2-KO macrophages and dendritic cells (DCs) produce more IL-6 in response to bacterial activation in vitro and in vivo. Thus, compared to wild-type (WT) mice, Tet2-KO mice show increased susceptibility to endotoxin-induced shock, DSS-induced colitis^40^ and *Salmonella* infections^41^. Tet2 transcription is induced by LPS activation, likely in a nuclear factor κβ (NF-κβ)-dependent way^42^. In vitro, Tet2 loss in BMDMs does not influence early LPS-induced gene activities but enhances arginase 1 (Arg1) mRNA level during later activation stages. Tet2 deletion does not dramatically alter alternative macrophage (M2) gene expression levels in response to IL-4 stimuli^39^. Tet2 expression is sustained in differentiated murine macrophages, and Tet2 deletion does not negatively alter markers of macrophage differentiation^42^. Resting Tet2-KO peritoneal macrophages reveal atypical induction of LPS-associated genes, and the Tet2-deficiency peritoneal macrophage features are linked to inflammation across the whole body^40^.

M1 macrophages are essential for tumor cell eradication^43^. Thus, modulating Tet2 expression levels in M1 macrophages might contribute to cancer-immune therapy (Fig. 1). Over a 20-week time-course, certain Tet2-KO strains show strong defects in myelopoiesis, leading to features similar to human CMML^7^. Acute inducible Tet2/Tet3 DKO in HSCs causes the rapid emergence of aggressive myeloid leukemia. Tet2 maintains the immunosuppressive functional role of tumor-infiltrating myeloid cells, promoting the advancement of melanoma^39^ and Tet2 deletion in myeloid cells reduces melanoma tumor burden in vivo. In tumor-associated macrophages (TAMs), Tet2 expression is activated along the IL-1R/MyD88 axis^39^. Myeloid-specific Tet2 loss causes higher numbers of tumor-infiltrating T cells^39^. During melanoma growth, Tet2 expression is enhanced in myeloid-derived suppressor cells (MDSCs) and TAMs^39^. Further, Tet2 preserves immunosuppressive gene expression levels in TAMs. Mice with myeloid cell-specific Tet2 deletion exhibit diminished tumor growth, and enhanced tumor-infiltrating T cells; moreover, T-cell depletion suppresses the attenuated tumor progression^39^. Whether Tet2 plays a similar or different role in myeloid cell-mediated tumor growth in cancers other than melanoma awaits further investigation.

Here, I have summarized our current understanding of the functional roles of Tet proteins in B cells, various types of T-cell subsets, and M1/M2 macrophages in both physiological and pathological conditions. Further study is essential to investigate how all Tet proteins and 5hmC/5mC modulate DNA modification, gene expression, and transcriptional networks in various types of innate and adaptive immune cells, and to dissect the underlying molecular mechanisms and potential for cancer-immune therapy in this field.

## Tet2 regulation at the cancer-immunity interface

**Fig. 2:**
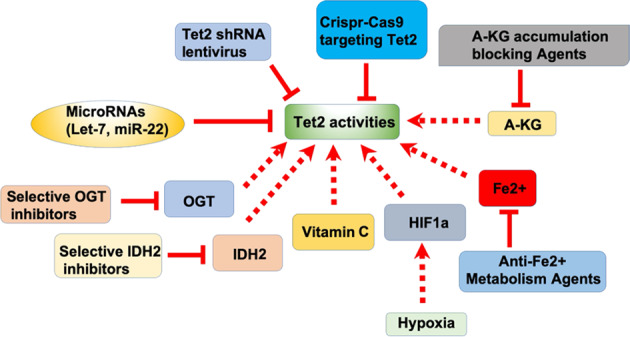
Potential therapeutic strategies related to cancer-immunity, based on interactors, specifically targeting Tet2 activities in immune and cancer cells. The Tet2 activities might be enhanced by vitamin C and hypoxia treatment through HIF-1a. The Tet2 activities might be halted by blocking a-KG accumulation, selective OGT inhibition, selective IDH2 inhibition, using anti-Fe^2+^ agent, or directly suppressed by microRNAs (e.g., Let-7 and miR-22), Tet2 shRNA lentivirus, and Crispr/cas9 targeting in cancer and immune cells.

Numerous regulatory mechanisms have either directly or indirectly been demonstrated to regulate Tet2 activities or expression levels in immune-cell subsets and cancer cells (Fig. 2). Mutated IDH1/2 enzymes, which are often found in tumors, produce the Tet2 protein inhibitor D-2HG, causing DNA hypermethylation^44^. Vitamin C interacts with the core catalytic domain of Tet2, directly boosting its catalytic activities^4^. Additionally, Tet2 expression can be directly altered at the post-transcriptional level^41,45^. Numerous microRNAs (miRs) post-transcriptionally inhibit Tet expression and certain miRs work together to control the expression of Tet genes. Tet2 genes contain conserved elements in the three prime untranslated regions (3′-UTR) predicted to be directly regulated by miR-22^46^. High-throughput screening has validated that the Tet2 expression level in hematopoietic cells is regulated by multiple miRs, including miR-7, miR-125b, miR-29b/c, miR-26, and miR-101^47^. Moreover, Tet2 can be directly and indirectly modulated by the miR let-7adf in macrophages^41^. O-GlcNAc transferase (OGT) transfers a single O linked-GlcNAc moiety to serine/threonine residues in multiple proteins^48^. OGT co-localizes with Tet2 in the genome, mainly at GC-rich promoters and transcriptional start sites (TSS) containing CpG islands, and reduces Tet2’s enzymatic activities by enhancing its nuclear export^49^. In the context of metabolic pressure, Tet2 can also be modulated by alpha-ketoglutaric acid (α-KG)^50^. Tet2 deletion through CRISPR-CAS9 causes globally enhanced DNA methylation at enhancers, thus blocking the estrogen responses^51^, indicating that Tet2 acts as a transcriptional coactivator in the estrogen responses. Fe^2+^ and HIF-1a also support to maintain Tet2 activities^52^. Thus, various cofactors (e.g., Fe2 + , HIF-1a and OGT) and noncoding RNAs (such as miRs) can affect Tet2 functional roles, constituting an additional level of regulation. Moreover, the epigenetic regulator CXXC5 and Tet2 are essential in the fight against viral infections. CXXC5 enrolls Tet2 to modulate TLR7/9-elicited IFN responses, and to influence DNA methylation, in human plasmacytoid DCs^53^. RUNX1 alters DNA demethylation by recruiting Tet2 and other enzymes with demethylation functional roles to its attaching sites in various hematopoietic cells^54^. All of these interactors, and other small-molecule activators of Tet2, would be helpful methods for stabilizing in vitro-generated immune cells or modified tumor cells for potential clinical utilization.

Although many Tet2 interactors have been identified in both immune and cancer cells, several principal questions are waiting for answers. For instance, how do these interactors assist Tet2 dysregulation-mediated cancer transformation as well as anticancer immune responses? Further work is needed to demonstrate the underlying molecular mechanisms. It will also be crucial to determine the relationship between Tet2 interactors in Tet2 dysregulation of the progression of non-hematopoietic cancers and blood cancers. Additionally, it is crucial to investigate whether and how these interactors influence 5hmC quantification, which is a helpful diagnostic and/or prognostic tool in cancer treatment. Manipulation of the activity of Tet2 interactors via small molecules or other modulators would be useful for future cancer-immune therapy.

## Targeting Tet2 for potential cancer-immune therapy

There may be several potential ways of specifically targeting Tet2 activity in immune-cell subpopulations and cancer cells (Fig. 2). In humans, functional loss of Tet2 plays an extensive potentiating role in immunotherapy against B-cell cancer, which utilizes T cells bearing the anti-CD19 chimeric antigen receptor (CAR). The insertional mutation of one copy of a Tet2 allele causes dysregulation of Tet2 activities, which apparently couples with the other Tet2 allele mutation, ultimately yielding strong antitumor properties to the expanded CAR-T cells^55^. Research on Tet2 loss in immune-cell responses and tumor development unveils the significance of DNA methylation in various biological activities, and in the advancement of hematological malignancies and solid tumors. With regards to potential clinical usage, it is hard to precisely target Tet2 for treating cancer progression, due to its inactivation in various cancer cell types. However, Tet2 is still expressed to certain levels in some immune-cell subsets and may be modulated in various manner (Fig. 2). For instance, fumarate hydratase (FH) and succinate dehydrogenase (SDH) inhibitors might be developed to prevent the global effectiveness of metabolic Tet2 inactivation in CD8^+^ T cells. Notably, the oncometabolite 2-hydroxyglutarate (2HG), generated by IDH mutation in immune cells, could also halt Tet2 activities in immune cells, (e.g., CD4^+^ T cells and M1 macrophages), which would trigger less-positive antitumor immune responses. Thus, it might also be valuable for future preclinical studies to investigate the restoration of Tet2 in these immune cells, for instance, by directly adding vitamin C or a-KG. Future work will focus on the accurate targeting of Tet2 in the context of different specific cell types, among both cancer and immune cells.

Investigations in both mouse models and human clinical samples have demonstrated that Tet2 loss is not essential to cause cancer, but rather influences cancer progression. This may be by reason for the behavior of Tet2 in various types of immune-cell subsets, and it might explain why some patients with Tet2 mutations stay healthy, whereas others develop either myeloid or lymphoid cancer. Future studies are needed to examine therapies targeting cytosine modifications, DNA methyltransferase inhibitors, and IDH inhibitors, which might be valuable for future cancer-immune therapy.

## Conclusions and current challenges

Collectively, Tet2 is critical to the cancer-immunity crosstalk, which is essential for both cancer development and anticancer immune responses (Fig. 1). It appears that the downregulation of 5hmC depends on certain specific cell types. It is expected that elevating Tet2 activities using specific compounds that enhance or potentiate Tet2 functional roles may be broadly useful in cancer treatment and for the modulation of certain anticancer immune-cell responses. To further interpret the underlying molecular mechanisms, it is critical to elucidate the functional roles of Tet2/5hmC regulators, and the different ways of modulating the acquisition/removal of 5hmC in various cell types. This will require additional insights into how Tet2 works, and how it is modulated in various types of mammalian cells, including both cancer and immune cells. The toughest challenge might be determining how to precisely modulate Tet2 in specific cells where it plays either positive or negative roles for antitumor therapy. Future work must also explore how to utilize Tet2 interactors to maintain positive antitumor immune responses and halt negative immune responses against cancers. There is still a lot to grasp regarding the physiological and pathological roles of Tet2 in other aspects (e.g., metabolism) in both immune and cancer cells. The elucidation of these aspects and molecular mechanisms would be an exciting avenue for further study.
